# Structure-based identification of small-molecule inhibitors that target the DIII domain of the Dengue virus glycoprotein E pan-serotypically

**DOI:** 10.1371/journal.pone.0311548

**Published:** 2024-10-25

**Authors:** Prakhar Agrawal, Hemant Arya, Ganesan Senthil Kumar

**Affiliations:** Integrative Structural Biology Laboratory, National Institute of Immunology, New Delhi, India; Arizona State University, UNITED STATES OF AMERICA

## Abstract

Dengue viral infection is caused by the Dengue virus, which spreads to humans through the bite of infected mosquitos. Dengue affects over half of the global population, with an estimated 500 million infections per year. Despite this, no effective treatment is currently available, however, several promising candidates are undergoing pre-clinical/clinical testing. The existence of four major serotypes is an important challenge in the development of drugs and vaccines to combat Dengue virus infection. Hence, the drug/vaccine thereby developed should neutralize all the four serotypes equally. However, there is no pan-serotype specific treatment for Dengue virus, thereby emphasizing the need for the identification of novel drug-like compounds that can target all serotypes of the Dengue virus equally. To this end, we employed virtual screening methodologies to find drug-like compounds that target the domain III of glycoprotein E. Most importantly, domain III of E protein is involved in viral fusion with the host membrane and is also targeted by neutralizing antibodies. Our study found two small molecule drug-like compounds (out of the 3 million compounds screened) having similar binding affinity with all four serotypes. The compounds thereby identified exhibit favourable drug like properties and can be developed as a treatment for Dengue virus.

## Introduction

Among different pathogens transmitted by mosquitos, Dengue virus transmission by *Aedes aegypti* stands distinct. According to the WHO report of 2023, there has been a ten-fold increase in Dengue virus infection between 2000 to 2019 with the disease spreading over to 129 countries putting more than 500 million people at risk [1]. Dengue virus has four serotypes (Dengue virus 1, Dengue virus 2, Dengue virus 3, and Dengue virus 4) and is highly prevalent in tropical and sub-tropical countries. Infection with any one serotype leads to long-term immunity to that said serotype but can lead to severe Dengue virus with secondary infections [2]. Given the high disease burden posed by the Dengue virus infection, it is critical to circumvent the infection using new therapeutics.

Previous studies have explored multiple approaches to treat Dengue virus infection that include monoclonal antibodies (mAbs), antivirals, and vaccines [3]. Some of the mAbs that have been shown to neutralize Dengue virus infection include humanized anti-Dengue virus mAb VIS513 by Visterra (Cambridge, Massachusetts) and AV-1 (AbViro LLC). However, these mAbs are currently in clinical trials (phase I) to understand their safety and pharmacokinetics [4–6]. On the vaccine front CYD-TDV (chimeric yellow fever virus–Dengue virus–tetravalent Dengue virus vaccine) aka Dengvaxia [7] (developed by Sanofi Pasteur), Dengusiil, developed by Serum Institute of India Pvt. Ltd [8], and QDENGA® or TAK-003 by Takeda Pharmaceuticals are safe in individuals [8], who were infected by Dengue virus previously, but their effect in serological negative populations is still under investigation [9]. Further, the loss of neutralizing epitopes as seen in SARS CoV2 [10] or inducement of severe Dengue virus due to potent neutralizing antibody (Antibody-Dependent Enhancement) [11,12] hampers the development of novel mAb/vaccine-based therapeutics.

With efficacy and safety being the major hurdles in the development of mAbs and vaccines, small molecules targeting Dengue virus provide an alternative strategy to combat Dengue virus infection. Previous studies have identified several antivirals like JNJ-64281802 [13] (NS4B inhibitor), Ivermectin [14] (host nuclear import receptor inhibitor), whereas AT-752 [15] (Guanosine nucleotide analog that targets NS5 RdRp function), and Doxycycline [16] (NS2B-NS3 inhibitor) to decrease viral load during Dengue virus infections. Among these molecules, Ivermectin failed to reduce viral load in patients [17], whereas AT-752 and JNJ-64281802 are in phase 1 and phase 2 clinical trials [18–21], respectively. Doxycycline is currently being studied in a double-blind randomized placebo-controlled clinical trial [22] for its safety and efficacy. Further, the approach that involves the repurposing of FDA-approved molecules to treat Dengue virus, compounds such as Curdlan sulfate [23], rolitetracycline [24], fucoidan [25], balapiravir [26], suramin [27], carnosine [28], policresulen [29], dasatinib [30], duramycin [31], luteolin [32], and imatinib [33] have *in vitro* antiviral activity. However, the absence of similar inhibitory activity against all four serotypes of Dengue virus limits further clinical trials [34–36]. Most importantly, most of the aforementioned small molecules that have been identified so far, target non-structural NS3 and NS5 proteins [34]. However, anti-Dengue virus compounds reported till now lack pan-serotype antiviral activity, has low efficacy, and toxicity [37].

Among the various stages of the Dengue virus life cycle, its entry into the host cell is crucial for viral replication and its inhibition can reduce the severity of Dengue virus infections, which remains unexplored as a drug target [36]. The viral entry is mediated by the structural glycoprotein E that consists of three structurally distinct domains: a central domain (DI), a dimerization domain (DII), and an immunoglobulin (Ig) like C terminal domain (DIII). Among these three domains, DIII, which is composed of 105 amino acids, is involved in receptor binding [38,39] and has been shown to reduce Dengue virus infectivity [40]. Interestingly, the DIII domain is one of the highly conserved domain across the Dengue virus serotypes and is also the target of many of the neutralizing antibodies [41,42], indicating its importance as a potential drug target.

In this study, we used computer-aided High Throughput Virtual Screening (HTVS), homology modelling, and Molecular Dynamics (MD) simulations to predict anti-DIII small-molecule inhibitors that target all the Dengue virus serotypes. Based on our findings, two molecules out of 3 million compounds examined met all criteria for acceptable ADME qualities, including strong and stable binding to domain III in all four serotypes. The two compounds identified can be investigated further as possible inhibitors of Dengue virus infection.

## Computational methods

### Preparation of protein

The crystal structures of domain III of Dengue virus type 1, 2, 3, and 4 (in complex with monoclonal antibody, mAb 4E11) were retrieved from Protein Data Bank (PDB) (PDB ids 3UZE, 3UZV, 3UZQ, and 3UYP, respectively). These structures were imported into the Maestro (Schrödinger Release 2024–1: Maestro, Schrödinger, LLC, New York, NY, 2024) and prepared for docking using protein preparation wizard [43]. During protein preparation, bond orders of the respective amino acids were assigned using the CCD database, disulfide bonds were created, hetero states for residues were generated using Epik at pH 7 ± 2, and hydrogen bond assignment was carried out at pH 7 using PROPKA. Further, any crystal waters beyond 3 Å from protein residues were deleted and restrained followed by energy minimization using OPLS/AA force field [43]. Subsequently, the DIII domain grid was generated using the Glide module in Schrödinger. For the generation of the grid, the domain III residues involved in interaction with mAb 4E11 were used (**S1 Table**). The interacting residues of the DIII domain with mAb 4E11 were identified using SPPIDER server (https://sppider.cchmc.org/) and PyMOL. The grid was generated around these residues that can accommodate any ligand of length ≤ 20 Å.

### Preparation of small molecules for docking

To identify the DIII domain-specific small-molecules, compounds from the following libraries were used: PubChem (https://pubchem.ncbi.nlm.nih.gov/), ChemDiv (https://www.chemdiv.com/catalog/ppi-modulators/protein-protein-interaction-library/), MMV pandemic box (https://www.mmv.org/mmv-open/pandemic-response-box/about-pandemic-response-box), Asinex (https://www.asinex.com/screening-libraries-(all-libraries)), and Life Chemicals (https://lifechemicals.com/screening-libraries/). Briefly, the SDF file of the compounds provided by the manufacturer was imported into the maestro and the LigPrep module was used to prepare small molecules for docking [43]. A possible ionization state was generated at pH 7 ± 2 using Epik. Following this, metal ions associated with the ligand molecules were removed and stereoisomers were generated (32 maximum) for each compound. During the generation of stereoisomers, the specific chirality as provided in their chemical structure was retained.

### Ligand-protein docking

The docking was performed using a Glide module [44] by omitting compounds with more than 500 atoms or 100 rotatable bonds. Three million compounds were screened using High-throughput Virtual Screening (HTVS) followed by Standard Precision (SP) and Extra Precision (XP) docking steps. While XP has maximal sampling and penalizes negative interaction such as steric clash, SP and HTVS don’t penalize negative interaction and do minimal sampling. At each stage, the top 10% of compounds were taken for the next round of screening.

### Binding free energy calculation

The binding free energy calculations were carried out using the Prime module of Schrödinger software [45]. The top 10% hits obtained from XP docking, were selected for *in silico* dG of binding score prediction. Briefly, the pose viewer file (.pv) post docking was taken as input for energy calculations. The dG of binding includes minimized energies, surface area energies, and polar and non-polar solvation energy. The energy is calculated as follows:

$$MMGBSA\;dG_{Bind}=dG_{complex}-dG_{receptor}-dG_{ligand}$$

where, component of total energy like coulombs, covalent binding energy, van der waals, lipophilic energy, generalized born electrostatic solvation energy are computed both for complex, protein, and ligand separately and the difference is reported in form of MMGBSA dG_bind_.

### ADME calculations

Integrating ADME (Absorption, Distribution, Metabolism, and Excretion) calculations in the identification of lead molecules can help in the evaluation of the lead compounds for their pharmacokinetic behaviour. This, in turn, will be useful for identifying potential false candidates (PAINS), optimization of lead compounds for lower toxicity and higher efficacy, and minimizing development risks. Therefore, the lead molecules were further subjected to the theoretical prediction of ADME parameters using the QikProp module of Schrödinger (Schrödinger Release 2024–1: QikProp, Schrödinger, LLC, New York, NY, 2024). These predictions are based on Quantitative Structure-Activity Relationship (QSAR) models, which employ mathematical models to correlate known compound structures with ADME properties and quantify the relationships between structural features and biological activities, assisting in the prediction of ADME parameters. The compounds exhibiting the following properties (as analyzed using QikProp module) were considered as good lead compounds for further analysis: (i) molecular weight of 600 to 700 Da, (ii) number of hydrogen bond donors (≤ 10), and acceptors (~30), (iii) the solubility parameter, denoted by QPlogPo/w, of around –2 to -6.5, (iv) the values for QPPCaco (model for gut-blood barrier) greater than 500 for high permeability and values lower than 25 for low poor cell permeability, and (v) the values of -3.0 to 1.2 for QPlogBB (model for blood-brain barrier), where negative values indicate a low transfer rate without active transport.

### Molecular Dynamics (MD) simulations

The structural stability of the top two lead compounds in complex with the DIII domains of Dengue virus serotypes was analyzed using GROMACS [GROningen MAchine for Chemical Simulations (GROMACS v2022.3)] [46] software. A total of eight independent 100 ns MD simulations were performed for the DIII domain of Dengue virus 1–4 serotypes in complex with the top two selected lead compounds (Compound IDs: 8296 and 10705) identified from HTVS. Automated Topology Builder v3.0 (https://atb.uq.edu.au/) webserver was used to generate ligand topology and force field parameters. The selected protein-ligand complexes were kept in the center of a cubic box with 10 Å sides and solvated the complexes using the Space Point Charge 216 (SPC 216) water model [47,48]. The bonded and non-bonded interactions were determined by the AMBER force field [46,49] and one Na^+^ ions was added to neutralize the systems. Initial minimization of the protein-ligand complexes was carried out using the Steepest-descent method (SD) [50,000 steps with a tolerance of 1000 kJ/mol] [50,51]. The modified Berendsen thermostat method was used to maintain the pressure (1 bar) and temperature (300 K) of the system [52]. Long-range electrostatics and covalent bond constraints were calculated using Particle Mesh Ewald (PME) methods and linear constraint solver (LINCS) algorithm, respectively [53,54]. To evaluate the stability of the system, the backbone Root Mean Square Deviation (RMSD), Root Mean Square Fluctuation (RMSF), radius of gyration (R_g_), potential energy, and hydrogen bonds between the protein and ligands were analyzed. The protein-ligand complexes were visualized using UCSF Chimera (https://www.cgl.ucsf.edu/chimera/) and plots were generated using XmGrace (http://plasma-gate.weizmann.ac.il/Grace/).

## Results and discussion

### Sequence and structural analysis of domain III of different serotypes of Dengue in association with mAbs

**Fig 1:**
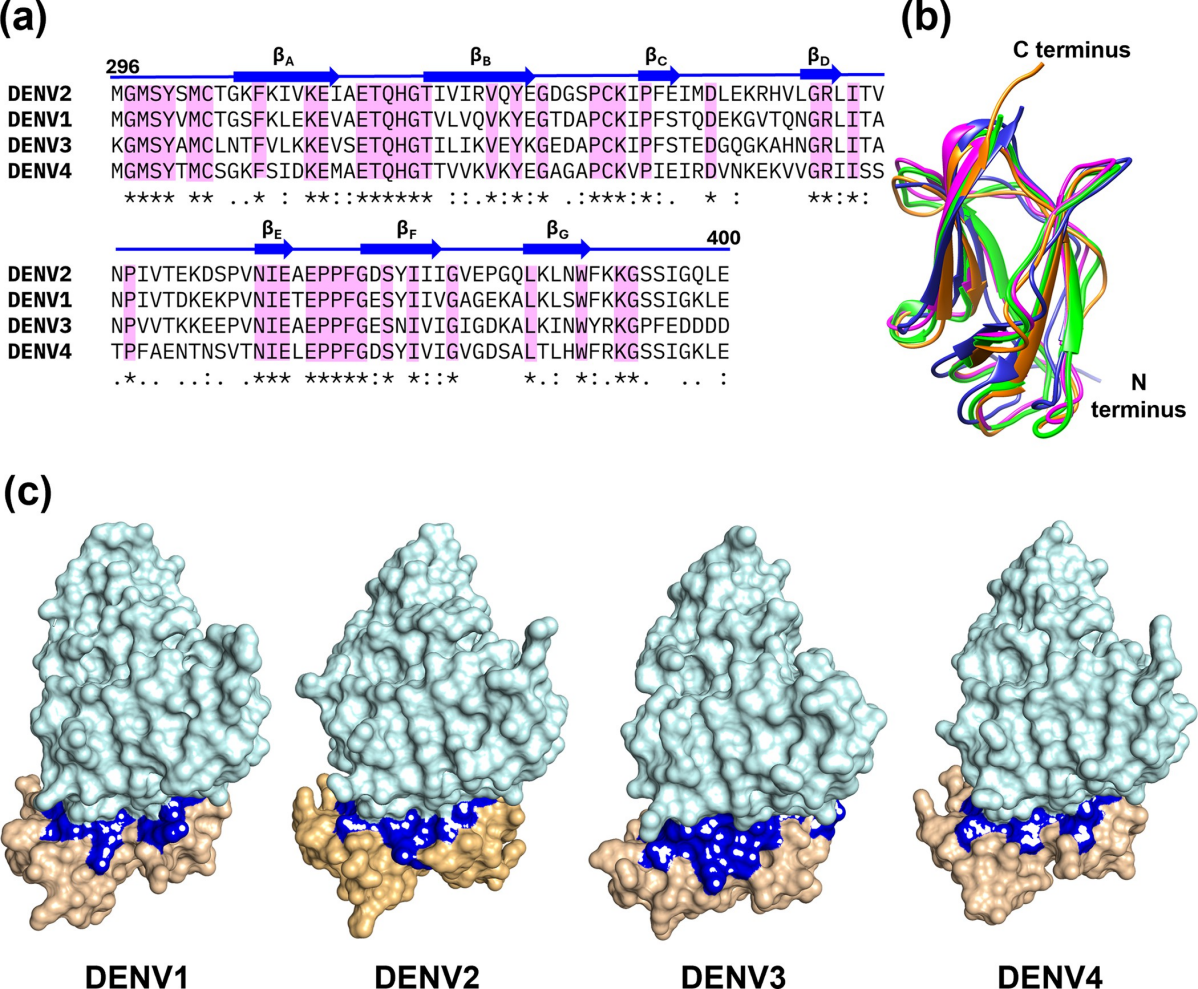
Conservation in domain III among Dengue virus serotypes, sequence and structural comparison, and its interaction with mAb 4E11. **(a)** Multiple sequence alignment of Domain III from Dengue virus serotypes (Dengue virus 1, Dengue virus 2, Dengue virus 3, and Dengue virus 4). The sequence alignment was done using ESPript (https://espript.ibcp.fr). **(b)** The 3D structural alignment of Domain III from Dengue virus serotypes, highlighting conserved structural elements. Dengue virus 1 is shown in magenta, Dengue virus 2 in blue, Dengue virus 3 in orange, Dengue virus 4 in green. **(c)** The interaction between Domain III and Monoclonal Antibody 4E11. The DIII structure is shown in light orange, mAb 4E11 in pale cyan and residues involved in the binding in blue.

Glycoprotein E of the Dengue virus (harboring three domains, namely domain I, II, and III), are involved in the endocytosis of the virus by binding through its domain III with diverse cell surface receptors facilitating the viral entry [55]. Importantly, most of the neutralizing antibodies identified so far, have been shown to interact with the DIII domain [41,56,57]. To test the conservation of DIII domain sequences across the serotypes, multiple sequence alignment (MSA) was performed. Dengue virus 2 shows a sequence identity of 65.7% with Dengue virus 1, 52.7% with Dengue virus 3, and 62% with Dengue virus 4 (**Fig 1A**). The sequence similarity stands at 79.6% for Dengue virus 2 and Dengue virus 1, 71.4% (Dengue virus 2 and Dengue virus 3), 77.8% (Dengue virus 2 and Dengue virus 4), respectively (**Fig 1A**). Moreover, the structural overlay of domain III from Dengue virus serotype 1 to 4 showed that, except for loops, there is a near-complete overlap of β sheets with a backbone RMSD of 0.487 Å indicating that the structures are highly similar (**Fig 1B**). Most of the available structures of domain III-antibody complexes in PDB is of Dengue virus type 2 with a few structures from Dengue virus type 1, Dengue virus type 3, and Dengue virus type 4 (**S2 Table**). To test whether the binding interface in the DIII domain with the antibodies is conserved across the serotypes, we mapped the interaction interface of mAbs 4E11, E106, Z004, 1A1D-2, 2H12, 3H5, 3E31, and 2D73 with the DIII domain of Dengue virus (**Figs 1C** and **S1**). Interestingly, these monoclonal antibodies like 4E11, 1A1D2, and 2D73 were observed to interact with domain III similarly, showing that the binding region of domain III is conserved across all Dengue virus serotypes [58,59] and is recognised by multiple antibodies. Further, the identification of the interaction interface of the mAb 4E11-DIII domain using PDBsum/SPPIDER/PymoL showed that the amino acids involved in the interaction were similar across the four serotypes (**S2 Fig**). Taken together, given its high sequence, structure, and interaction interface conservation across the serotypes, the DIII domain of glycoprotein E is a viable target for the identification of small molecules that could potentially inhibit the Dengue virus viral entry. Furthermore, the identified compounds will be able to overcome the antibody dependent enhancement (ADE) effects, which is due to binding of the neutralizing antibodies with the DIII domain. Therefore, developing small molecule inhibitors that bind to the DIII domain will be an efficient strategy for combating Dengue virus infection.

### Docking of small molecules from different libraries led to the identification of compounds with similar docking scores against the DIII domain of all four serotypes

**Fig 2:**
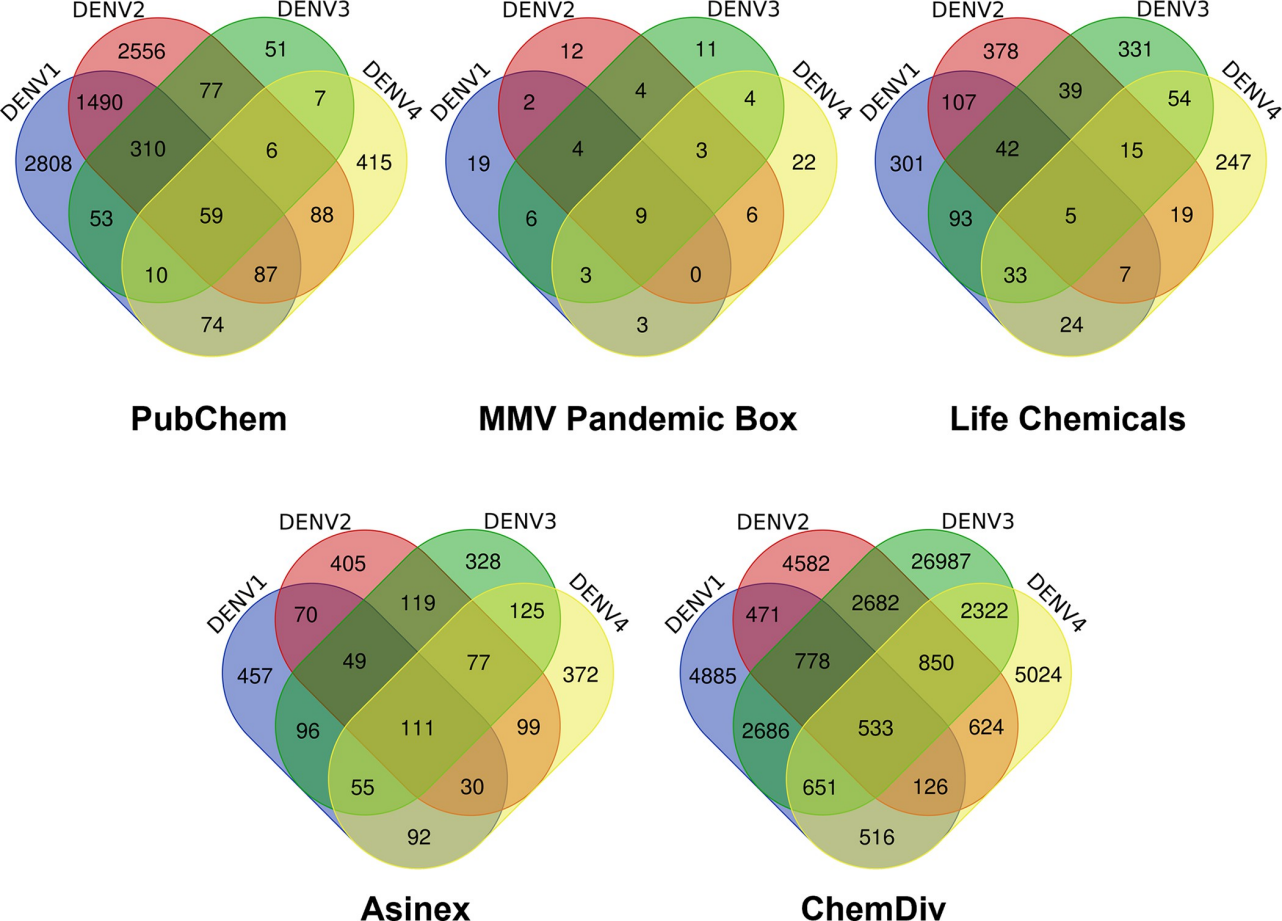
Identification of small molecules through high-throughput virtual screening. The Venn diagram illustrating the distribution of compounds identified across different Dengue virus serotypes (Dengue virus 1, Dengue virus 2, Dengue virus 3, and Dengue virus 4). The overlapping regions indicate compounds shared between two or more serotypes, while unique regions represent compounds exclusive to a particular serotype.

To identify a novel set of compounds that can prevent Dengue virus internalization in host cells, 3 million compounds from various libraries such as PubChem, ChemDiv, Asinex, LifeChemicals, and MMV were docked to the domain III using the Glide module. The compounds from PubChem, MMV, and LifeChemicals were categorized as "small molecules," whereas compounds from Asinex and ChemDiv were classified as "Protein-Protein modulators". The compounds were then ranked based on docking score (XP) and dG of binding for each serotype from each library and molecules that were common in all four serotypes were selected for further analysis (**Fig 2**). Based on this, nearly 717 compounds (59 PubChem, 9 Pandemic, 5 LifeChemicals, 111 Asinex, and 533 ChemDiv) were found to be common hits in all four serotypes (**S1 File**). As seen in **Fig 2**, the number of compounds binding specifically to Dengue virus 1 (2808) and Dengue virus 2 (2556) was relatively high as compared to Dengue virus 3 (51) and Dengue virus 4 (415) with 59 molecules common to all four serotypes in the case of PubChem compound library docking. The compounds from the MMV pandemic box, LifeChemicals, and Asinex compound library docking showed a similar number of compounds unique to all serotypes (**S1 File**). In the case of ChemDiv compounds docking, 26987 compounds were found to be unique to Dengue virus 3, followed by Dengue virus 4 (5624), Dengue virus 1 (4885), and Dengue virus 2 (4582). The high number of compounds in ChemDiv (that are specific to modulate/prevent protein-protein interactions) targeting Dengue virus 3 might be due to subtle structural/sequence differences among the serotypes. The molecules identified as common in all serotypes were found primarily belong to classes like peptidomimetics (amino acids, peptides, and analogues), amines, and pyrimidines, benzoxazepines, indoles, piperazines, and pyrazoles.

### Selection of potential pan-serotype small molecule inhibitors

**Fig 3:**
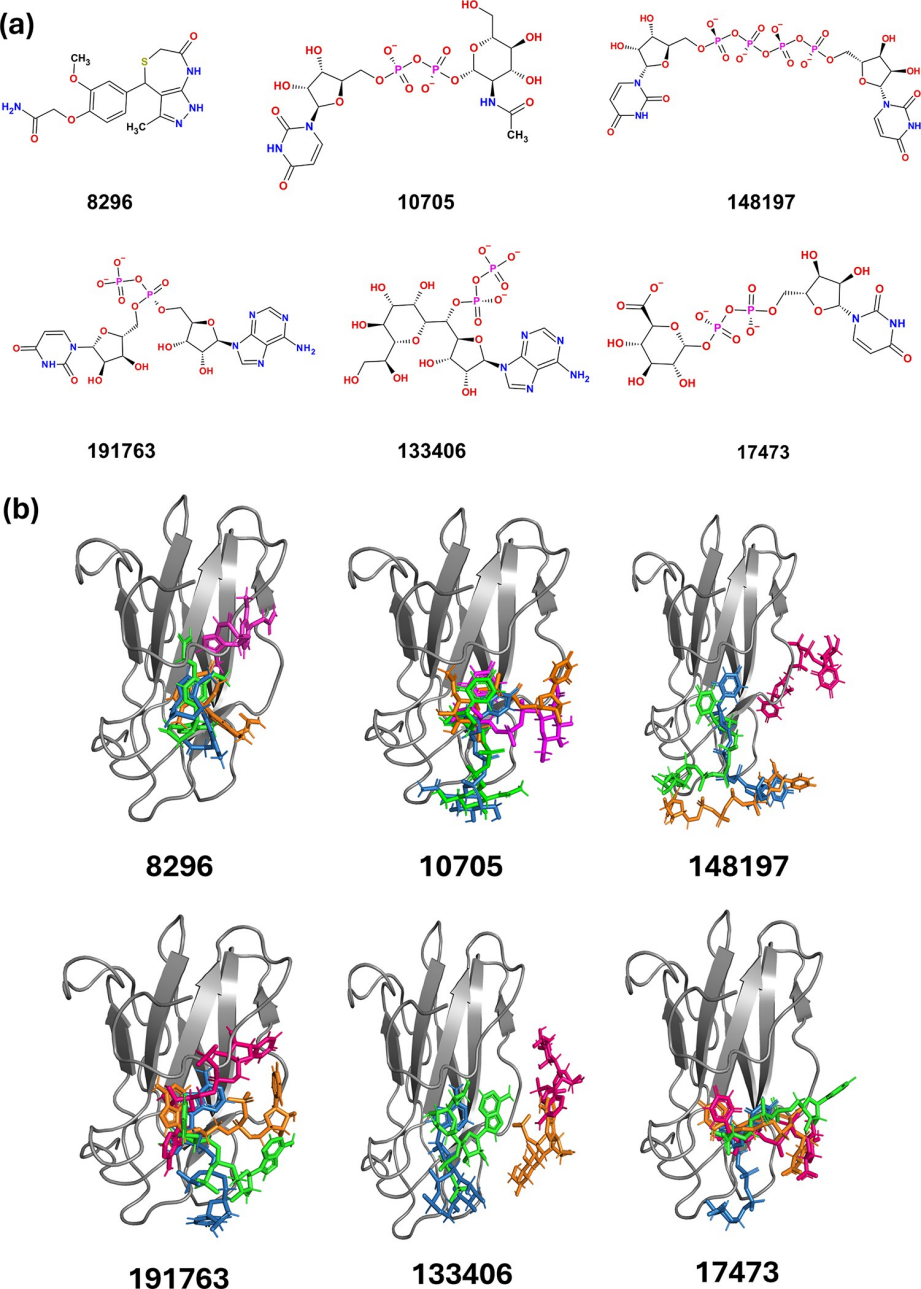
The chemical structures of lead molecules, and the structural relationships among these potential antiviral agents for Dengue virus. **(a)** The molecular structures of lead compounds identified as top binders to the Domain III (DIII) of the Dengue virus E protein for each serotype. The compounds belong to two main classes: Nucleotide analogues (148197, 191763, 133406, 10705, 17473) and phenol ether (8296). **(b)** Structural superimposition of the lead molecules identified from DIII binding for Dengue virus 1 (magenta), Dengue virus 2 (blue), Dengue virus 3 (orange), and Dengue virus 4 (green). Note identified small molecules bind at similar region across the serotypes.

To identify the compounds that are highly specific to all the serotypes, the identified 717 compounds were further classified based on the docking scores for each serotype. The docking (XP) score for the selected small molecules ranged from -1.5 kcal/mol to -8.9 kcal/mol, whereas the score for protein-protein modulators ranged from -1.8 kcal/mol to -6.4 kcal/mol. Further, dG of binding for the small molecules was in range of -9.93 kcal/mol to -66.66 kcal/mol whereas protein-protein modulators dG of binding was in range of -4.35 kcal/mol to -71.27 kcal/mol. The top 20 molecules in each serotype were selected and tabulated based on the docking score and dG of binding (**S1 File**). Of these twenty compounds, six of them were found to be common across the four serotypes. Interestingly, five of the six compounds that interacted specifically with all the four serotypes were nucleotide analogues, with the other one belonging to the class of phenol ether (**Fig 3A**).

As shown in **Table 1**, the six lead compounds identified had a varying but more or less close docking score and dG of binding for Dengue virus serotypes 1, 2, 3, and 4. In the case of Dengue virus 1 all compounds showed docking scores in range of -4 to -6 kcal/mol, though differed in dG of binding. For instance, compounds 10705 and 17473 have similar docking scores but differing dG of binding (**Table 1**). Similarly, in the case of Dengue virus 2, 17473 showed lowest docking score (-8.269 kcal/mol) however, dG of binding was lowest for 133406 (-46.27 kcal/mol). A similar observation was seen in the case of Dengue virus 3 and Dengue virus 4. Upon comparing the docking scores of compounds in different serotypes, it is observed that compound 17473 had lowest docking score (**Table 1**) in Dengue virus 2, whereas compounds 10705, 133406, 148197, 191763, and 8296 showed docking score < -4 kcal/mol in all four serotypes. Upon examining the dG of binding, which reflects the strength of binding, the compound 10705 shows lowest score in Dengue virus 1, compound 17473 Dengue virus 4 showed higher dG of binding (-27.78 kcal/mol) when compared with other serotypes (**Table 1**). The compound 133406 showed the lowest dG of binding in Dengue virus 2 compounds, while the compounds 191763 and 148197 showed the lowest dG of binding in Dengue virus 3 and Dengue virus 4, respectively. On the other hand, the compound 8296 had dG of binding in range of -24.59 kcal/mol to -32.69 kcal/mol in different Dengue virus serotypes.

**Table 1:** Docking and dG of binding scores for the compounds identified common across serotypes. All the values of docking scores and MMGBSA dG binding scores are reported in kcal/mol.

Library	Code	Dengue virus 1		Dengue virus 2		Dengue virus 3		Dengue virus 4	
		XP Docking score	MMGBSA dG Binding score	XP Docking score	MMGBSA dG Binding score	XP Docking score	MMGBSA dG Binding score	XP Docking score	MMGBSA dG Binding score
**PubChem**	10705	-6.06	-51.46	-6.884	-36.79	-5.722	-25.64	-6.293	-21.17
	17473	-5.95	-38.6	-8.269	-36.54	-8.269	-35.15	-6.492	-27.78
	133406	-6.08	-20.23	-7.504	-46.27	-7.504	-29.49	-7.225	-16.19
	148197	-6.469	-36.13	-7.649	-35.88	-7.649	-16.13	-7.892	-33.52
	191763	-6.405	-36.48	-6.687	-41.36	-6.687	-48.79	-6.129	-23.82
**ChemDiv**	8296	-4.463	-29.06	-4.55	-24.59	-5.101	-32.69	-4.107	-26.19

The identification of compounds using *in silico* methods, docking score and dG of binding are equally important [60]. The scoring function underlines the importance of hydrogen bonds, ionic interactions, and hydrophobic interactions during docking. Hence, comparing the compounds across all the four serotypes, the compound 8296, from ChemDiv showed comparable binding energy in the range of -24.59 kcal/mol to -32.69 kcal/mol, whereas average docking score across all four serotypes were found to be -4.55 kcal/mol. Similarly, compound 10705 had near identical docking scores in all serotypes with dG of binding in the range of -21 kcal/mol to -51 kcal/mol. Other lead compounds identified showed different scores in different serotypes (**Table 1**). The differences and similarities in the docking scores and dG of binding can be explained based on differential interaction of compounds to the domain III. Further, it is evident that compounds 10705 and 8296 are having similar docking site in all serotypes (**Fig 3**), whereas in other compounds the binding site residues are relatively different in amongst serotypes.

### Interaction of compounds with E DIII of Dengue virus

**Fig 4:**
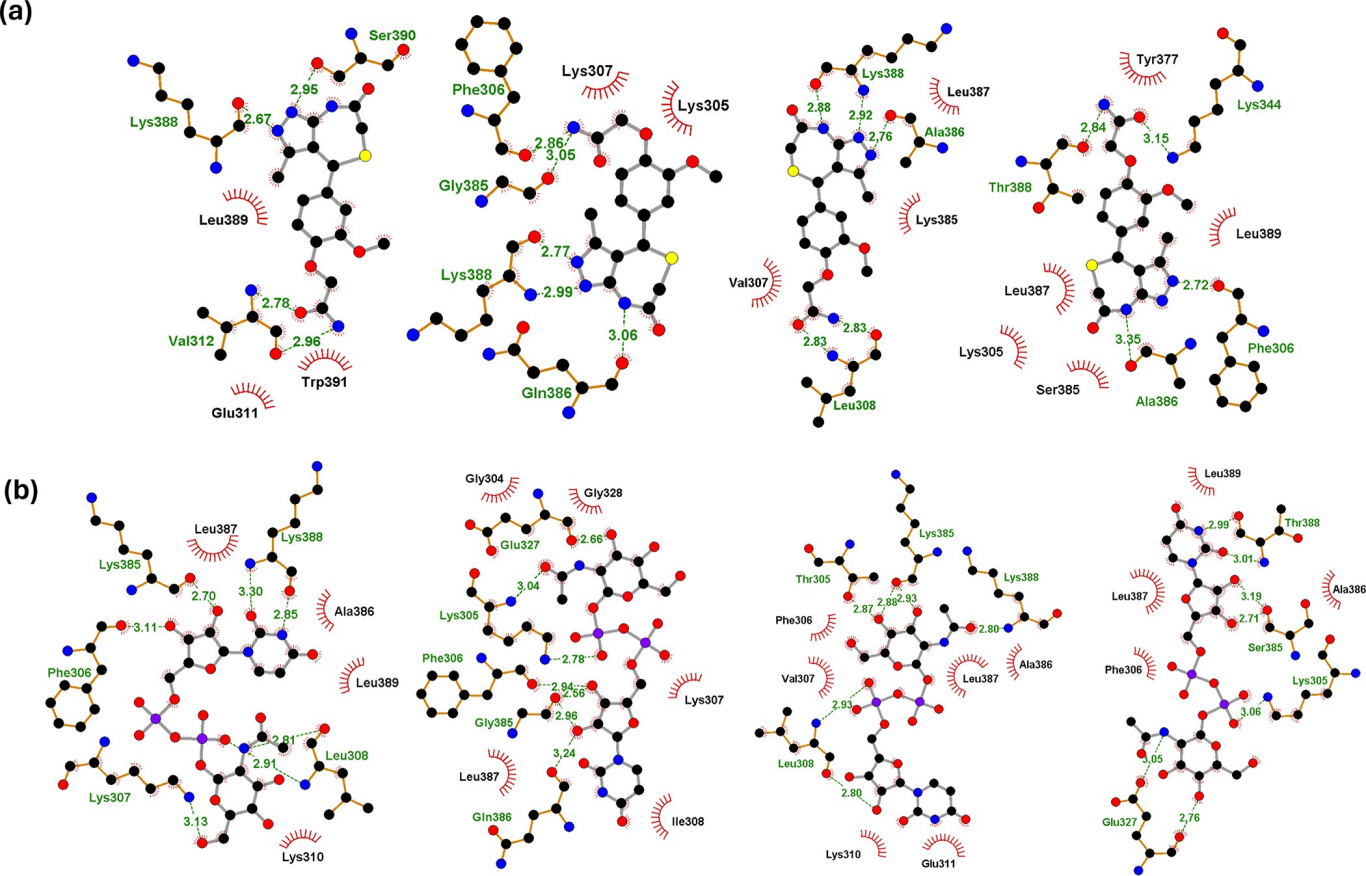
Molecular docking results of the DIII domain of Dengue virus 1–4 (left to right) with lead compounds (a) 8296 (b) 10705 at the active site of interaction. Atoms are coloured as follows: Nitrogen is blue, oxygen is red, carbon is black, sulphur is yellow, phosphorus is purple, and hydrophobic residues are brick red. Similarly, grey bonds represent ligand bonds, orange: Non-ligand bonds, olive green: Hydrogen bonds, red: Salt bridges, and brick red: Hydrophobic interactions.

The binding of the identified six Domain III binders (**Fig 3A**) in different serotypes are shown in **Fig 3B**. Looking at the interface of the protein-small molecule complex, compounds 17473, 10705, 191763, and 8296 have relatively similar binding sites across all the serotypes. In the case of compound 148197, its binding site in Dengue virus 1 differs from Dengue virus 2, 3, and 4. Similarly, compound 133406 binding sites in Dengue virus 3 and Dengue virus 4 differed from Dengue virus 1 and Dengue virus 2. Considering the importance of hydrogen bonding in stabilizing this interaction, the number of hydrogen bond between domain III and the compounds were compared. In the case of Dengue virus 2, Gly385 was found to be a conserved residue involved in the backbone hydrogen bonding with all six lead molecules. The other residues like Lys305, Gln386, Glu327, Lys388, Phe306, Lys361, Lys307, and Ile388 were seen to form hydrogen bonds with one or more than one compounds like 10705, 133406, 17473, and 8296 (**Fig 4, S3 and S4 Figs**). In the case of Dengue virus 1, no conserved residue was found to be involved in hydrogen bonding in all small molecule-domain III complex; however, residues like Lys388, Lys385, Leu308, Val312, Ser390, Glu311, Gln323, and Lys310 were found to be conserved amongst different pair of compounds like 10705 and 8296 (Lys388), 10705 and 17473 (Lys385 and 308), 191763 and 8296 (Val312 and Ser390), 133406 and 148197 (Glu311, Gln323 and Lys310) (**Fig 4, S3 and S4 Figs**). Similarly, in case of Dengue virus 3, Leu308 was common in 10705,191763 and 8296; Lys385 in 10705,148197 and 191763; Lys388 and Ala386 in 10705 and 8296. Further, when different compounds were compared for common interacting residues in Dengue virus 4, residue 305 (Lys) was found common in all compounds, Thr388 in 10705, 8296, 133406, 191763 and Phe306 common in 148197,17473,191763, and 8296. When residues involved in the hydrogen bond between compounds and domain III were compared across the serotypes, Phe306 was conserved in all four, whereas Lys388 was common in Dengue virus 2 and Dengue virus 1, and Lys305 was common in Dengue virus 2 and Dengue virus 4 (**S1 Table**). The interaction of compounds in all serotypes with DIII in manner similar to that of mAb 4E11, highlights the potential of the identified molecules in preventing the domain III interaction with the cell surface receptors.

### ADME profiling shows lead molecules have a drug like properties

To evaluate the druggability of the compounds identified, the QikProp module of Schrödinger was used to perform ADME analysis, and the results are summarized in **Table 2**. Based on this ADME properties, the compound 8296 showed zero violations of the Lipinski rule of five (**Table 2**) and had a molecular weight of 362.40 Da with four and eight hydrogen bond donor and acceptor respectively. Also, it had human oral absorption at 54.64% along with 0.167 and 31.113 values for QPlogPo/w and QPPCaco, respectively. The other five compounds namely 148197, 191763, 133406, 10705, and 17473 had molecular weight in the range of 580 Da to 790.311 with hydrogen bond donor of more than five and acceptors of more than ten. Although, the Lipinski rule of five have been conventionally used as the standard criteria set to predict the druggability of compounds, there are exceptions where few of the molecules violate rule of five specifically rules related to molecular weight and hydrogen bond acceptors/donors and yet are FDA approved drugs [61–64]. Hence, all the six compounds show promising potential as novel anti-Dengue virus drugs despite having few exceptions in the Lipinski’s rule. In summary, the compounds exhibit favourable properties, with limited absorption and distribution, and low CNS permeability. These data provide valuable insights into the compounds’ potential as drug candidates.

**Table 2:** The table below presents predicted ADME (Absorption, Distribution, Metabolism, and Excretion) properties for a set of compounds. The data includes parameters related to the Rule of Five, absorption, distribution, and central nervous system (CNS) permeability.

Library	Code	Rule Of Five	Absorption		Distribution			CNS Permeability		
			HOA	%HOA	QPPCaco	QPPMDCK	QPlogKhsa	CNS	QPlogBB	PSA
PubChem	148197	3	1	0	0	0	-3.964	-2	-8.608	427.584
	191763	3	1	0	0.04	0.015	-2.352	-2	-4.748	307.502
	133406	3	1	0	0.001	0	-2.628	-2	-6.267	339.096
	10705	3	1	0	0.009	0.005	-2.797	-2	-5.471	331.058
	17473	3	1	0	0.001	0	-2.661	-2	-5.904	339.954
ChemDiv	8296	0	2	54.642	31.113	32.656	-0.526	-2	-1.768	139.103

%HOA: % Human Oral Absorption.

### Molecular dynamics simulations

**Fig 5:**
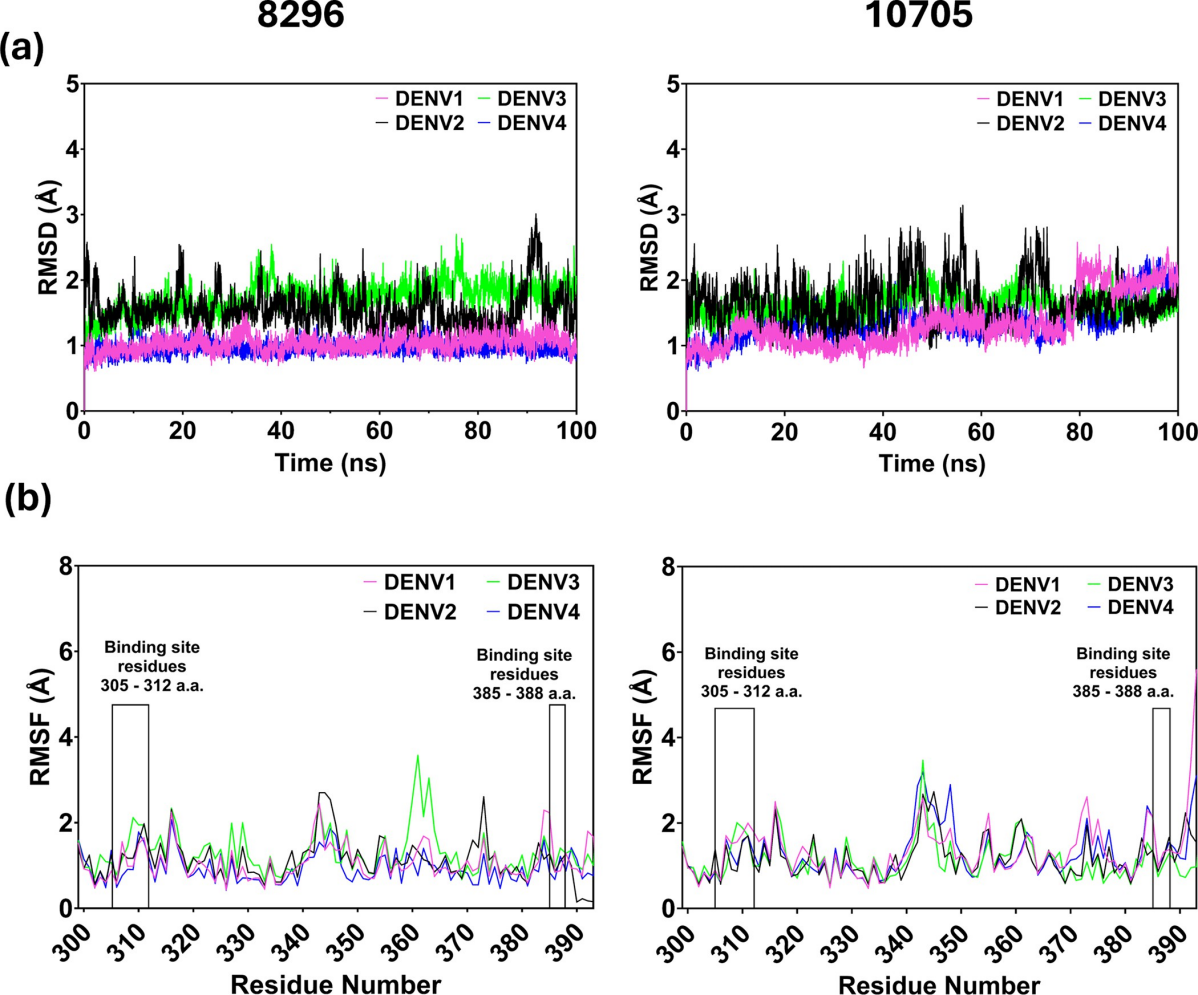
MD simulations of domain III in presence of compound 8296 and 10705. **(a)** The C^α^ backbone RMSD values over time, reflecting the deviation of the simulated structure from the starting reference structure for compound 8296 (left panel) and 10705 (right panel). **(b)** The RMSF values for individual residues, highlighting the degree of fluctuation or flexibility during the simulation. Higher RMSF values indicate more flexible regions, while lower values suggest relatively stable regions. Binding site residues show remarkably low RMSF fluctuations suggesting stable interaction between the compound and the protein. The DIII domain of Dengue virus 1, 2, 3, and 4 are shown in magenta, black, green, and blue, respectively. The interacting region is highlighted in a box.

Among the top six molecules, two molecules i.e., molecules 8296 and 10705 had more or less similar docking score and dG of binding for all serotypes. Further, upon comparing structure of compounds, 8296 has distinct structure belonging to phenol ether class, whereas other compounds are nucleotide analogues. Among these nucleotide analogues, 10705 showed docking score and dG of binding more or less similar to other nucleotide analogues **(Table 1)** identified and bears resemblance to both 17473 and 191763 in terms of functional groups on either side of dihydrogen diphosphate bond (**Fig 3A**). Most importantly, both compounds bind at the similar interface across all the serotypes and hence were taken for molecular dynamics simulation studies to assess the stability of their interaction with the DIII domain. To assess the Dengue virus serotypes (1, 2, 3, 4) in complex with selected two leads (8296 and 10705), 100 ns MD simulations were performed using GROMACS software and the stability of the compounds were analyzed by RMSD, RMSF, potential energy, radius of gyration and H-bond interactions. The average deviation of the target’s backbone structure with respect to its initial structure is described by the RMSD, whereas the Rg assesses the compactness of the system. The RMSF indicates the average fluctuations of each residue with respect to its starting position. The average H-bonds that form between a protein and ligand are analyzed on the H-bond interactions. The backbone C^α^ RMSDs of the small molecules 8296 and 10705 with Dengue virus 2 serotypes vary between 1.0 and 3.5 Å, whereas other Dengue virus serotypes (1, 2, 3) vary between 0.8 to 2.2 Å. The overall RMSD plot indicates that the complexes remain stable during the MD run (**Fig 5A**). Comparably, the RMSF graph of the chosen protein-ligand complexes (Dengue virus 1–4–8296/10705) showed little variation, except for the loop area (340–365 AA), N–terminal, and C–terminal regions (**Fig 5B**). There were RMSF values less than 3.0 Å. Furthermore, both compounds (8296 and 10705) were seen to form stable interactions with the binding site residues of Dengue virus serotypes: Leu308, Val312, Lys388, Ser390 (Dengue virus 1), Phe306, Lys388, Gly385, Gln386 (Dengue virus 2), Leu308, Ala386, Lys388 (Dengue virus 3), Lys305, Phe306, Lys344, Ala386, Thr388 (Dengue virus 4), as evident from the reduced RMSF changes in the aforementioned residues (ranging from 1.2 to 1.5 Å) (**Fig 5B**).

The selected protein-ligand complexes were compact and stable during the MD run, as seen from the R_g_ plot (**S5A Fig**), indicating the stability of the complexes. The potential energies of the selected complexes varied between -3.3 x 10^−5^ to -5.5 x 10^−5^ kJ/mol (**S5B Fig**). The hydrogen bonding interactions observed during 100 ns simulation for the compound 8296 showed relatively higher number of hydrogen bonds in the case of Dengue virus 2 followed by Dengue virus 1, Dengue virus 4 and Dengue virus 3 (**S5C Fig**). On comparing the average number of hydrogen bonds during simulation, the compounds 8296/10705 were seen to form one—two hydrogen bonds with the binding site residues of Dengue virus serotypes (**S5C Fig**). Based on the above results, it is conclusive, during the MD run, both the compounds (8296 and 10705) were stable in their binding pocket of the targets (Dengue virus 1–4), and forms stable interactions with Dengue virus serotypes with fluctuations for Dengue virus 1 (**S6 Fig**). To further validate the stable binding of compounds to their respective targets, binding free energy calculation post molecular dynamics and RMSF of DIII residues in vicinity of compounds was analysed. The average binding free energy during M.D. (**S7 Fig**) for compound 8296 was found to be -32.40 kcal/mol (Dengue virus 1), -28.49 kcal/mol (Dengue virus 2), -51.71 kcal/mol (Dengue virus 3), -39.34 kcal/mol (Dengue virus 4). Similarly, for compound 10705, average binding energy was -25.50 kcal/mol (Dengue virus 1), -29.29 kcal/mol (Dengue virus 2), -25.11 kcal/mol (Dengue virus 3), -40.68 kcal/mol (Dengue virus 4). Similarly, RMSF of protein residues in binding cavity showed RMSF less than 1.2 Å (**Fig 5B**) which was less considering the binding region to be highly flexible. Thus, based on the MD simulation study, we conclude that the two compounds (8296/10705) were making stable interactions with Dengue virus serotypes (1, 2, 3, 4) and could be potential leads for further investigations.

## Conclusion

Dengue illness poses a concern to tropical and subtropical countries. The changing environment and rising vector population in previously Dengue virus -free areas have caused a socioeconomic crisis with far-reaching implications. With no Dengue virus drugs and no viable mAb/vaccine, there is an urgent need to identify new therapeutics to combat this disease. In this light, our findings demonstrate that the domain III, which was previously untapped for drug discovery, is a promising target for identifying compounds that can be used as building blocks for the development of strong pan-serotype Dengue virus inhibitors. Further, our studies have identified two potential inhibitors that needs to be tested using *in vitro* and *in vivo* studies to test their efficiency as anti-Dengue virus compounds.

## Supporting information

S1 Fig The surface representation of domain III of dengue (depicted in light orange) in complex with various monoclonal antibodies (E106, Z004, 1A1D-2, 2H12, 3H5, 3E31, 2D73) to highlight the identical interaction interface. The highlighted region (depicted in blue) denotes the interface where the Domain III interacts with the respective Ab.
(TIF)

S2 Fig Interaction of domain III (DIII) of dengue virus envelope protein with monoclonal antibody 4E11 evaluated using PDBsum. The figure integrates visualizations derived from PDBsum, providing a detailed view of the structural details of the DIII and mAb 4E11 complex.
(TIF)

S3 Fig Interaction of molecules with DIII of DENV1, 2, 3, and 4(left to right) (a) 148197. (b) 191763.
(TIF)

S4 Fig Interaction of molecules with DIII of DENV1, 2, 3, and 4 (left to right) (a) 133406. (b) 17473.
(TIF)

S5 Fig Potential energy, radius of gyration, and hydrogen bond analysis from molecular dynamics simulation of domain III in presence of compound 8296 and 10705. (a) shows the Radius of Gyration (Rg) values over time. Rg measures the compactness of the compound-DIII complex, with higher Rg values indicating a more extended or flexible structure and lower values suggesting a more compact and stable conformation. (b) shows the potential energy profile throughout the simulation. Potential energy is a measure of the system’s total energy and is indicative of the stability of the compound-DIII interactions. Consistent potential energy values suggest equilibrium, while fluctuations may indicate dynamic events. (c) The number of hydrogen bond forming with respect to time during molecular dynamics simulation of compound 8296 and 10705 in complex with domain III of E protein.
(TIF)

S6 Fig Ligand fit plot illustrating the dynamic behaviour of the compound within its binding site during a 100 ns simulation. The x-axis represents the simulation time in ns, while the y-axis represents the ligand’s RMSD in Å. The DIII domain of DENV 1, 2, 3, and 4 are shown in magenta, black, green, and blue, respectively.
(TIF)

S7 Fig Changes in dG of binding during 100 ns simulation. Complex structures were taken for every 5 ns and energy calculation was done using Prime MMGBSA tool of Schrödinger software.
(TIF)

S1 Table Residues involved in domain III interaction with mAb 4E11 in different dengue serotypes. (DOCX)

S2 Table Domain III of E protein in association with antibodies; PDB and antibody id. (DOCX)

S1 File Docking, MM-GBSA, and QikProp scores for the molecules identified as binders to Domain III of dengue. (XLSX)
