# Supplementary figures and images for: Structure-based identification of small-molecule inhibitors that target the DIII domain of the Dengue virus glycoprotein E pan-serotypically
Source: PLoS One. 2024 Oct 25;19(10):e0311548. doi: 10.1371/journal.pone.0311548 (PMC11508475; doi:10.1371/journal.pone.0311548)

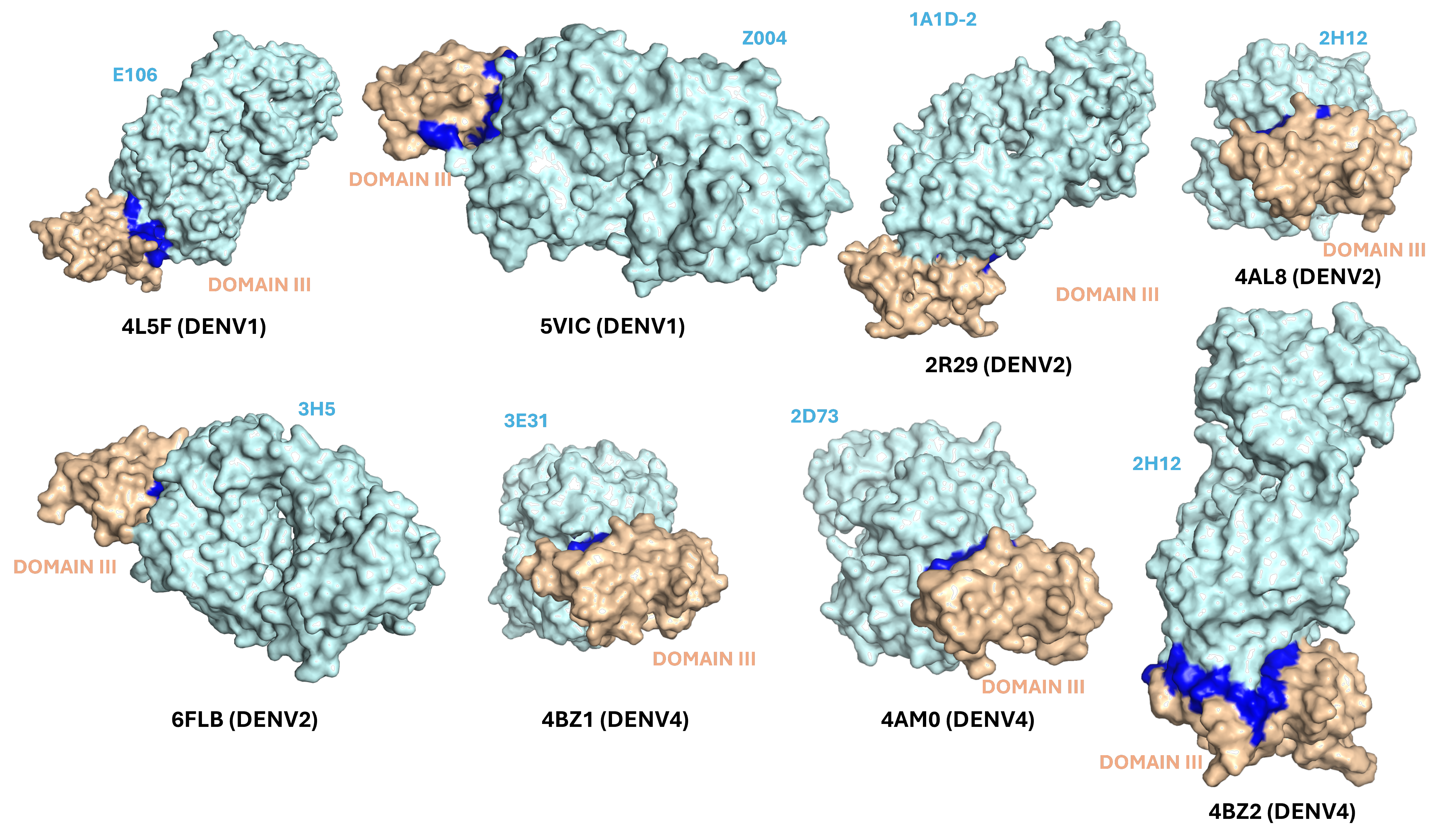

Supplement: S1 Fig — The highlighted region (depicted in blue) denotes the interface where the Domain III interacts with the respective Ab. (TIF) [file pone.0311548.s001.tif]

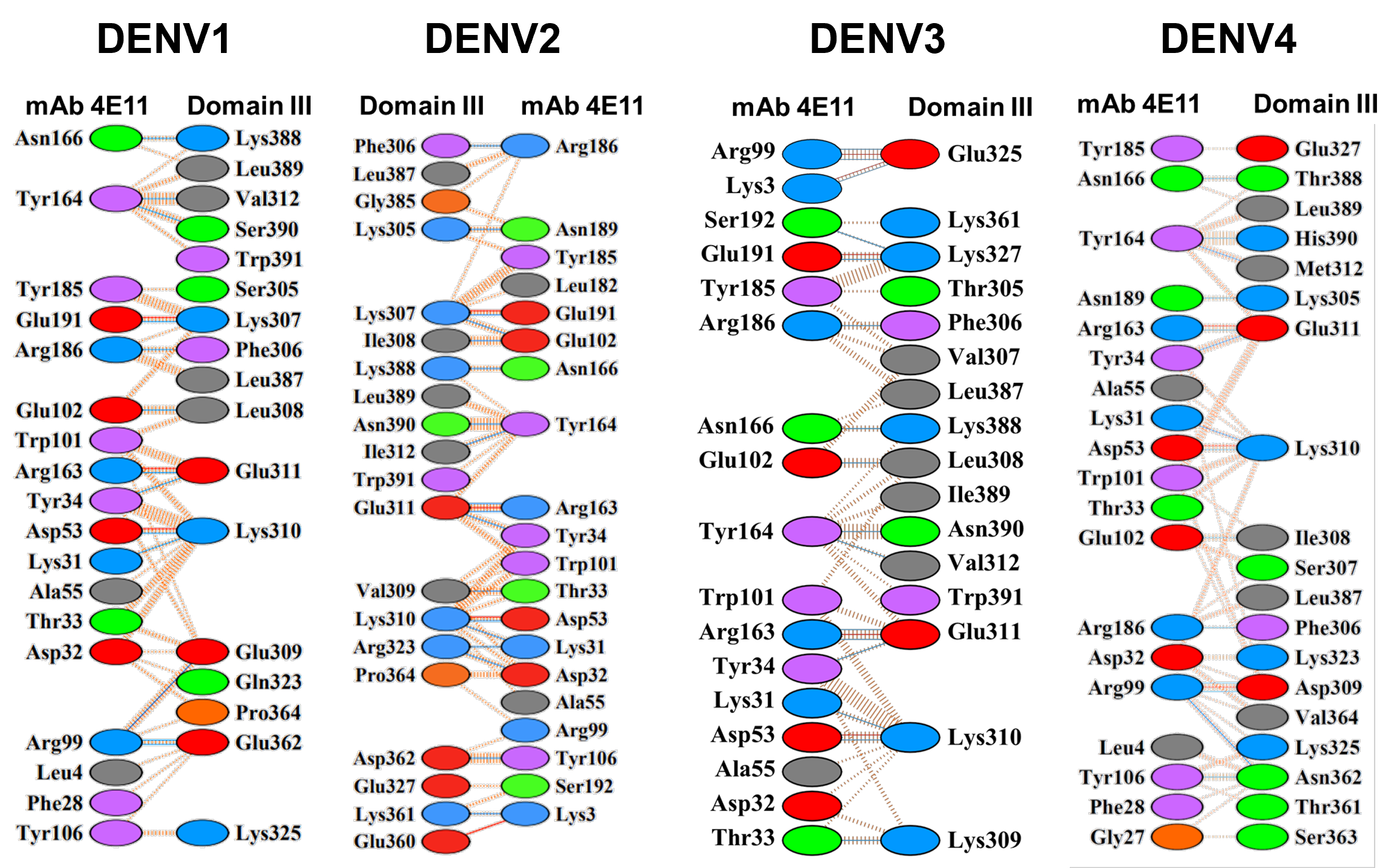

Supplement: S2 Fig — The figure integrates visualizations derived from PDBsum, providing a detailed view of the structural details of the DIII and mAb 4E11 complex. (TIF) [file pone.0311548.s002.tif]

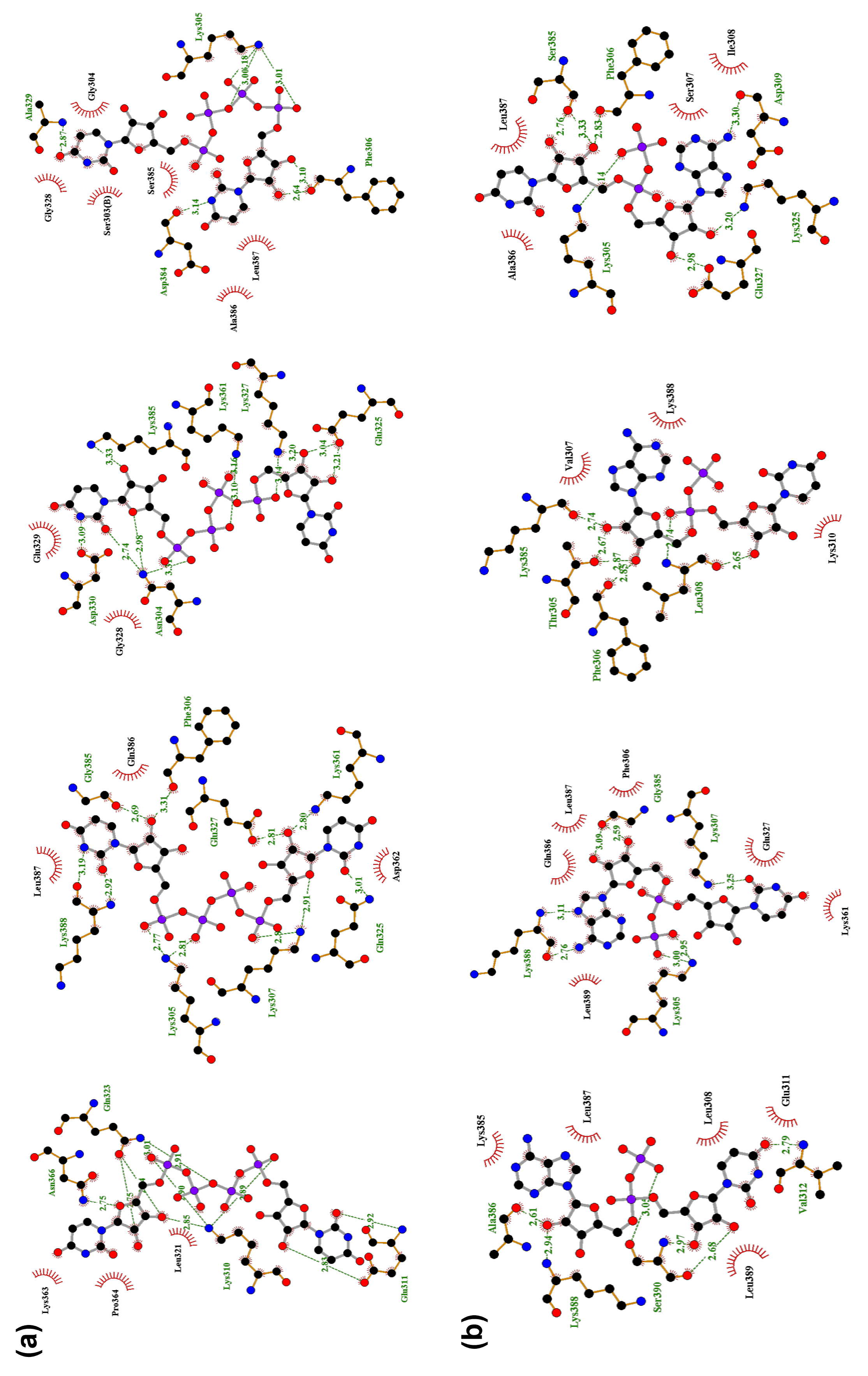

Supplement: S3 Fig — Interaction of molecules with DIII of DENV1, 2, 3, and 4(left to right) (a) 148197. (b) 191763. (TIF) [file pone.0311548.s003.tif]

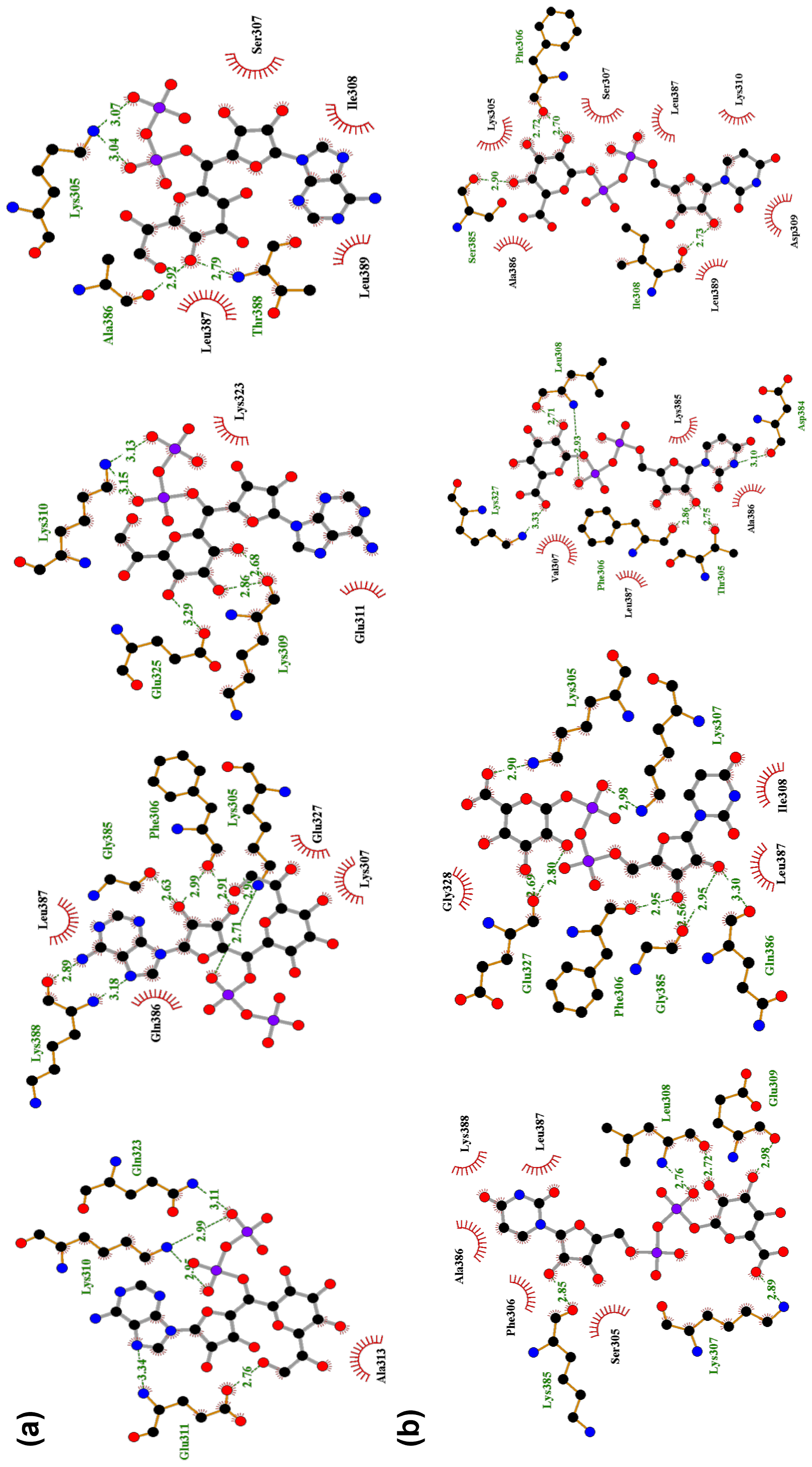

Supplement: S4 Fig — Interaction of molecules with DIII of DENV1, 2, 3, and 4 (left to right) (a) 133406. (b) 17473. (TIF) [file pone.0311548.s004.tif]

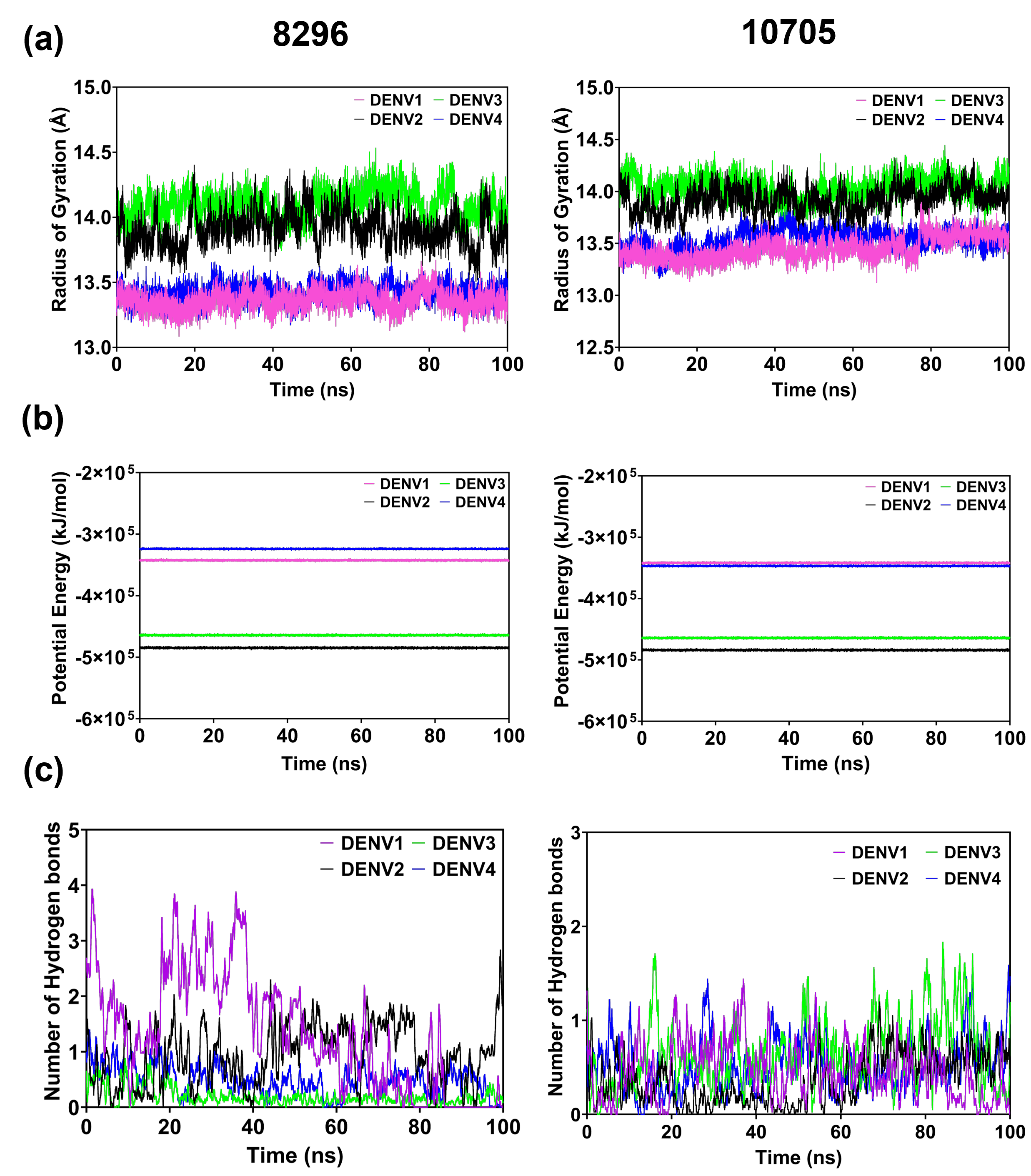

Supplement: S5 Fig — (a) shows the Radius of Gyration (Rg) values over time. Rg measures the compactness of the compound-DIII complex, with higher Rg values indicating a more extended or flexible structure and lower values suggesting a more compact and stable conformation. (b) shows the potential energy profile throughout the simulation. Potential energy is a measure of the system’s total energy and is indicative of the stability of the compound-DIII interactions. Consistent potential energy values suggest equilibrium, while fluctuations may indicate dynamic events. (c) The number of hydrogen bond forming with respect to time during molecular dynamics simulation of compound 8296 and 10705 in complex with domain III of E protein. (TIF) [file pone.0311548.s005.tif]

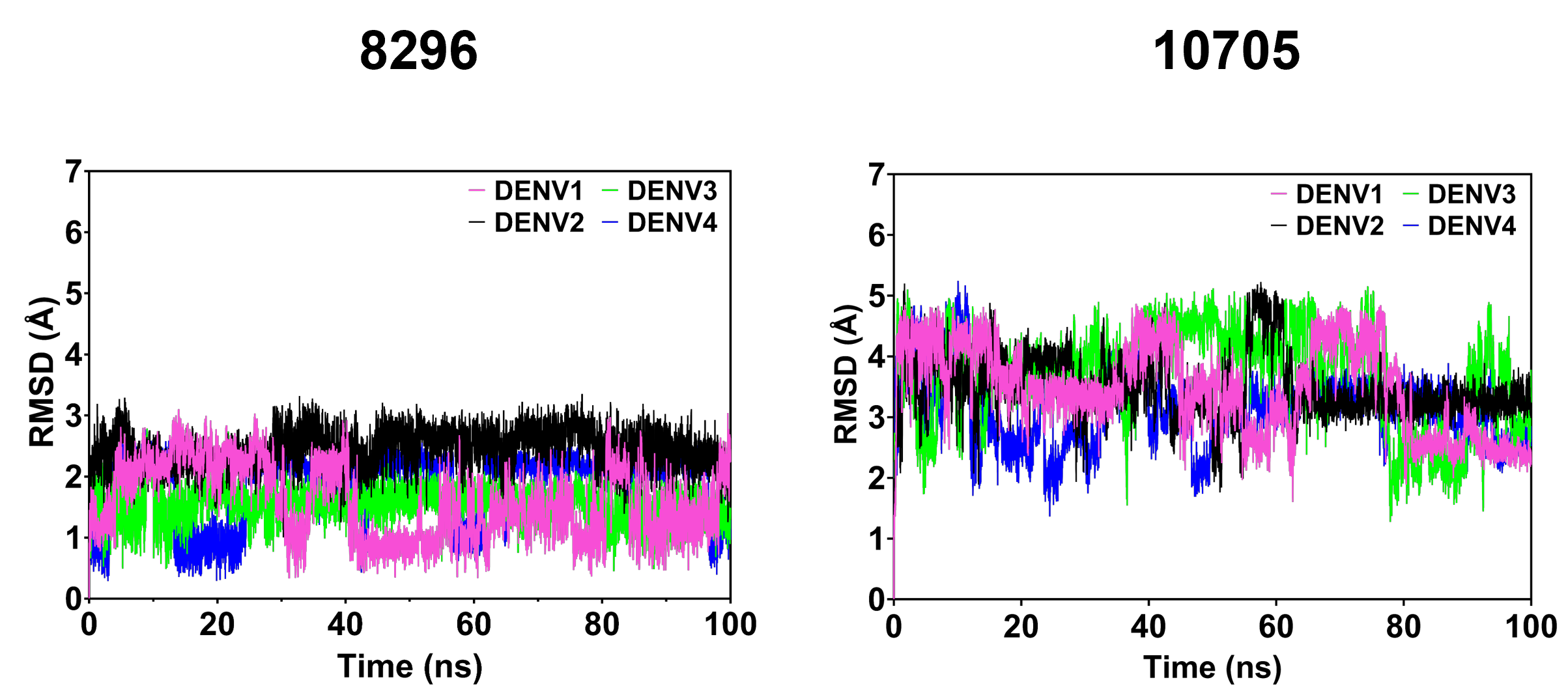

Supplement: S6 Fig — The x-axis represents the simulation time in ns, while the y-axis represents the ligand’s RMSD in Å. The DIII domain of DENV 1, 2, 3, and 4 are shown in magenta, black, green, and blue, respectively. (TIF) [file pone.0311548.s006.tif]

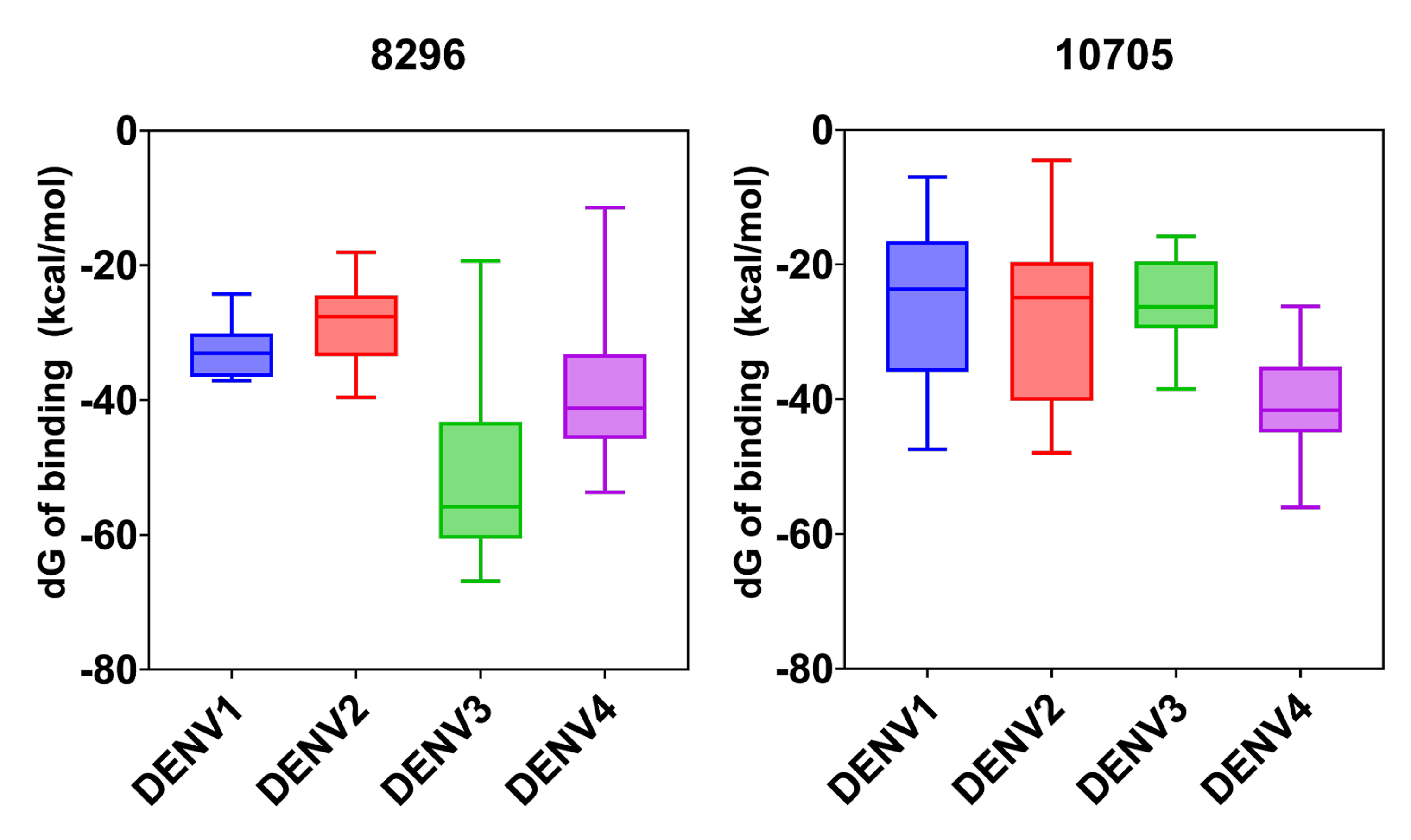

Supplement: S7 Fig — Complex structures were taken for every 5 ns and energy calculation was done using Prime MMGBSA tool of Schrödinger software. (TIF) [file pone.0311548.s007.tif]
