# Supplementary material for: Structure-based identification of small-molecule inhibitors that target the DIII domain of the Dengue virus glycoprotein E pan-serotypically
Source: PLoS One. 2024 Oct 25;19(10):e0311548. doi: 10.1371/journal.pone.0311548 (PMC11508475; doi:10.1371/journal.pone.0311548)
Supplement: S1 Table — (DOCX) [file pone.0311548.s008.docx]

**Supplementary Table 1: Residues involved in domain III interaction with mAb 4E11 in different dengue serotypes**

| **DENV serotype** | **DIII residues involved in Hydrogen bonding with mAb 4E11** |
| --- | --- |
| **DENV1** | F306, K307, L308, E309, K310, E311, V312, Q323, K325, E362, P364, L387, K388, L389, S390, W391 |
| **DENV2** | K305, F306, K307, I308, V309, K310, E311, I312, R323, E360, K361, D362, P364, G385, L387, K388, L389, N390, W391 |
| **DENV3** | T305, F306, V307, L308, K309, K310, E311, V312, E325, K327, K361, L387, K388, I389, N390, W391 |
| **DENV4** | K305, F306, S307, I308, D309, K310, E311, M312, K323, K325, E327, T361, N362, S363, V364, L387, T388, L389, H390 |
