# Supplementary material for: Structure-based identification of small-molecule inhibitors that target the DIII domain of the Dengue virus glycoprotein E pan-serotypically
Source: PLoS One. 2024 Oct 25;19(10):e0311548. doi: 10.1371/journal.pone.0311548 (PMC11508475; doi:10.1371/journal.pone.0311548)
Supplement: S2 Table — (DOCX) [file pone.0311548.s009.docx]

**Supplementary Table 2: Domain III of E protein in association with antibodies; PDB and Antibody id.**

| **S.No.** | **PDB id** | **Antibody** | **DENV serotype** |
| --- | --- | --- | --- |
|  | 3UZQ | 4E11 | DENV1 |
|  | 4L5F | E106 | DENV1 |
|  | 5VIC | Z004 | DENV1 |
|  | 6DFJ | Z021 | DENV1 |
|  | 2R29 | 1A1D-2 | DENV2 |
|  | 3UZV | 4E11 | DENV2 |
|  | 4AL8 | 2H12 | DENV2 |
|  | 4ALA | 2H12 | DENV2 |
|  | 6FLB | 3H5 | DENV2 |
|  | 6FLC | 2C8 | DENV2 |
|  | 3UZE | 4E11 | DENV3 |
|  | 3UYP | 4E11 | DENV4 |
|  | 4AM0 | 2H12 | DENV4 |
|  | 4BZ1 | 3E31 | DENV4 |
|  | 4BZZ | 2D73 | DENV4 |
